# SINGLE PORT LAPAROSCOPIC CHOLECYSTECTOMY: TECHNICAL ASPECTS AND RESULTS

**DOI:** 10.1590/0102-672020180001e1388

**Published:** 2018-08-16

**Authors:** Murillo de Lima FAVARO, Silvio GABOR, Ruy Francisco Pimentel PEDROSO, Ligia RIBEIRO, Otto Mauro ROSA, Marcelo Augusto Fontenelle RIBEIRO-JUNIOR

**Affiliations:** 1 General Surgery Department, University of Santo Amaro; 2 Private Clinic, São Paulo, SP, Brazil

**Keywords:** Laparoscopy, Cholecystectomy, laparoscopic, Cholecystectomy, Colecistectomia, Colecistectomia laparoscópica, Laparoscopia

## Abstract

**Background:**

The search for less traumatic surgical procedures without compromising efficacy and safety, together with the technological advances and greater experience of the teams, led to the development of operative techniques with increasingly smaller incisions, the so-called “minimally invasive surgeries”.

**Aim:**

To evaluate the technical aspects and results of single port cholecystectomy.

**Method:**

Were analyzed 170 patients between 18-74 years submitted to videolaparoscopic cholecystectomies by single port, regardless of elective or urgent indication, without restriction of patient selection.

**Results:**

Among the 170 operations, 158 were exclusively performed by single port, and the conversion rate was 7% (inclusion of other accessory trocars or conversion to multiportal). Conversion to open surgery occurred in three cases (1.76%). The mean surgical time was 67.97 min, showing a marked decrease when was reached close to 50 cases and a stabilization after 100 surgeries. The overall complication rate was 10%, with minor complications such as: incisional pain, hematomas, granulomas, port access hernias (9.41%).

**Conclusion:**

Single port cholecystectomy can, after standardization and surgical team training, be a safe surgical procedure associated with a recognized aesthetic advantage.

## RESUMO

**Racional:**

A busca de procedimentos cirúrgicos menos traumáticos, esteticamente melhores, sem comprometimento da eficácia e segurança, em conjunto com os avanços tecnológicos e maior experiência das equipes, levaram ao desenvolvimento de técnicas operatórias com incisões cada vez menores, as denominadas “cirurgias minimamente invasivas”.

**Objetivo:**

Avaliar os aspectos técnicos e resultados da colecistectomia por portal único.

**Método:**

Foram analisados 170 pacientes entre 18-74 anos submetidos à colecistectomias videolaparoscópicas por portal único, independente da indicação eletiva ou de urgência, sem restrição de seleção dos pacientes.

**Resultados:**

Das 170 operações, 158 foram exclusivamente realizadas por portal único, sendo que a taxa de conversão foi de 7% (inclusão de outros trocárteres acessórios ou conversão para multiportal). Já a conversão para laparotomia ocorreu em três casos (1,76%). A média de tempo cirúrgico foi de 67,97 min, mostrando decréscimo acentuado quando chegou-se perto dos 50 casos, e estabilização após a centésima operação. A taxa de complicação global foi de 10% sendo que em sua maior parte foram complicações leves como: dor incisional, hematomas, granulomas, hérnias no portal de acesso (9,41%).

**Conclusão:**

A colecistectomia por portal único consegue, após padronização técnica e treinamento da equipe cirúrgica, ser procedimento cirúrgico seguro, associado à vantagem estética reconhecida.

## INTRODUCTION

The search for less traumatic surgical procedures, aesthetically better, without compromising efficacy and safety, together with the technological advances and greater experience of the surgical teams, led to the development of operative techniques with increasingly smaller incisions, the so-called “minimally invasive surgeries”^19^ ^,^ ^22^ ^,^ ^24^ ^,^ ^30^. They are currently the modality of choice for many of the surgical interventions. Its benefits are numerous: lesser response to surgical aggression, shorter recovery time and return to personal and professional activities, lower rates of postoperative pain, infections, incisional hernias, as well as smaller and more esthetic operative scars^10^ ^,^ ^18^ ^,^ ^19^ ^,^ ^20^ ^,^ ^22^ ^,^ ^24^ ^,^ ^30^. Minimally invasive operations began in 1909, when Hans C. Jacobaeus performed the first laparoscopy in humans and in 1918, was adopted the use of pneumoperitoneum. In 1987 Philippe Mouret in Lyon - France performed the first laparoscopic cholecystectomy in the world. Thomas Szego in 1990 began laparoscopic surgery in Brazil. Since then, technological advances have boosted the development of minimally invasive surgeries^18^ ^,^ ^23^ ^,^ ^24^.

 The introduction of natural orifice transluminal endoscopic surgery (NOTES) by Kalloo stimulated interest in an even less invasive approach than conventional multiportal laparoscopic surgery (MPLS)^14^.

Thus, the umbilical scar was chosen as a single surgical access route, with the use of a port with multiple channels or several single ports introduced by the same incision, representing an option between MPLS and NOTES. In 1997, Navarra et al. report the first cholecystectomy performed by a single port “Single Incision Surgery” in humans^19^.

The single-port operation carries the advantages of NOTES as far as the cosmetic and less invasive approach is concerned, but without compromising gastrointestinal organs or other viscera. Studies indicate that the complication rates with the single port in relation to MPLS are not increased^3^ ^,^ ^6^ ^,^ ^8^ ^,^ ^11^ ^,^ ^18^ ^,^ ^25^ ^,^ ^28^ ^,^ ^29^ ^,^ ^30^.

In addition, by using conventional laparoscopic instruments, single-port operation provides vision of the abdominal cavity similar to MPLS, which makes the procedure more familiar to the surgeon, despite increasing the degree of difficulty at the beginning of the training by working with parallel clamps, leading to an eventual clash between them, a decrease in triangulation and difficulty in traction and contra-traction maneuvers. As for NOTES, specific training with a longer learning curve is necessary for the use of apparatus and endoscopic techniques^2^ ^,^ ^30^.

The main advantage of single port operation compared to MPLS, until the present moment, is the cosmetic aspect^5^ ^,^ ^11^ ^,^ ^15^ ^,^ ^17^ ^,^ ^18^.

The objective of this study was to evaluate the technical aspects and results of single port cholecystectomy.

## METHOD

Were analyzed 170 patients between 18-74 years submitted to videolaparoscopic cholecystectomies by single port between February 2011 and July 2015, regardless of elective or urgent indication, without restriction of patient selection. All procedures were performed by the same surgical team, following the same technical standards, within several hospitals in the city of São Paulo, SP, Brazil.

Data such as gender, age, BMI, surgical time (time of video recording of the operation added to the timed time of surgical access and its closure), wound closure, devices used, complications and need for conversion to multiportal or laparotomic technique, were obtained and analyzed.

With the patient in the horizontal dorsal position with the surgeon placed between the legs, the assistant with the videocamera on the left and the scrub nurse on the right of the patient, the trocar was placed under direct vision through an umbilical incision of about 20 mm and a pneumoperitoneum was performed between 10-12 mmHg. Conventional videolaparoscopy instruments with optics of 30° degrees, 10 mm and 42 cm in size and straight needle nylon suture were used to expose the gallbladder. Four single-port trocar models, mostly SILS^®^ (Medtronic-Covidien) and Gelpoint^®^ (Applied) were used, due to the better adaptation. Initially the closure of the umbilical aponeurosis opening was done with prolipropylene 0 with continuous suture, and a proglactin 910 thread with separate stitches and inverted node has been routinely used. Anesthesia was infiltrated with 7.5% bupivacaine in the surgical wound during closure in all cases. All the patients were followed so far for more than 18 months. 

### Statistical analysis

Statistical analysis was performed using ANOVA, chi-square test, Yates correction, Pearson’s correction and correlation test. Statistical significance was assumed when the p value was less than 0.005 (p<0.05).

## RESULTS

There were 75% of female and 25% of male patients treated. Only 29% of the patients were within the range of normal BMI; 56% were overweight and 15% were obese.

**FIGURE 1:**
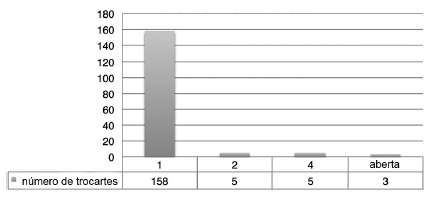
Number of trocars used

Of the 170 surgeries, 158 were exclusively performed by single port, with conversion rate of 7% (inclusion of other accessory trocars or conversion to MPLS, Figure 1). The conversion rate for laparotomy was 1.76% (n=3), with the main indication of difficulty in identifying the critical safety vision.

**FIGURE 2:**
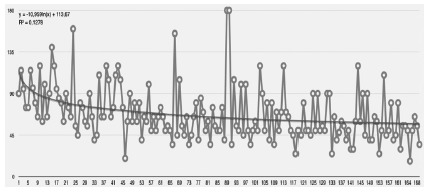
Surgical time throughout the operations

The mean surgical time was 67.97 min, showing a marked decrease when the study approached the 50 cases and stabilized after the hundredth operation (Figure 2). The maximum surgical time was 180 min and a minimum of 17 min.

Four brands of single port trocars were used, one from Triport^®^, four from Single Site^®^, 29 Gelpoint^®^ and 136 SILS^®^.

**FIGURE 3:**
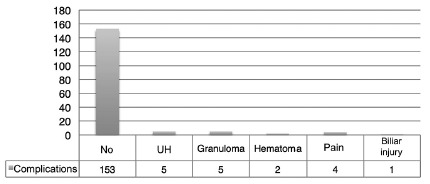
Number and type of complications

The overall complication rate was 10%, with minor complications such as: incisional pain, hematomas, granulomas, port access hernias (9.41%). In one case (0.59%) a grade D biliary lesion on the Dindo-Clavien complication scale was observed. In this case the conversion to laparotomy was performed and a primary choledochal repair with the placement of a Kehr drain was done^21^ ^,^ ^22^. This single patient presented satisfactory postoperative evolution, without clinical losses (Figure 3).

No statistically significant correlation was found between age, BMI and surgical time, therefore, they are statistically independent results (Pearson’s test and Correlation, Table 1).

**TABLE 1:** Influence of BMI on single port operations

		Age	BMI
BMI	Corr (r)	9,5%	
	p	0,216	
Operative time	Corr (r)	0,5%	1,2%
	p	0,953	0,877

Finally, the ANOVA test was used to compare the means of quantitative variables among subgroups of qualitative variables. There is a mean BMI difference for the complications of granuloma and umbilical hernia. Patients with granuloma had a mean BMI lower than that without granulomas. The mean BMI of the umbilical hernia patients was 31.4%, compared with 26.4% of those without a hernia (Table 2).

**TABLE 2:** Correlation between complications and BMI

BMI		Mean	Median	SD	CV	Min	Max	N	CI	p
Granuloma	No	26.7	27	3.8	14%	17	39	165	0.6	0.041
	Yes	23.2	22	1.8	8%	22	26	5	1.6	
Pain	No	26.5	26	3.8	14%	17	39	166	0.6	0.374
	Yes	28.3	28	2.6	9%	26	31	4	2.6	
Biliar lesion	No	26.6	26	3.8	14%	17	39	169	0.6	0.366
	Yes	30.0	30	-x-	-x-	30	30	1	-x-	
Umbilical hernia	No	26.4	26	3.7	14%	17	39	165	0.6	0.004
	Yes	31.4	30	4.7	15%	26	38	5	4.1	
Hematoma	No	26.6	26.5	3.8	14%	17	39	168	0.6	0.055
	Yes	21.5	21.5	0.7	3%	21	22	2	1.0	
General complication	No	26.5	27	3.7	14%	17	39	153	0.6	0.637
	Yes	27.0	26	4.8	18%	21	38	17	2.3	

## DISCUSSION

The results of the present study show a higher proportion of the population of operated women, due to the characteristic of the disease itself. Likewise, the BMI of the most predominantly operated population was overweight, BMI between 25 and 30^12^.

The MPLS and laparotomy conversion index was 7%. Differently from the literature; no predictive factors, gender, age or BMI were found affecting conversion rates^3^. The conversion to laparotomy was required in three cases (1.76%), which is much lower than that found in the literature when compared to MPLS conversion indexes. Since this technique is still considered the gold standard operation, this index of 7% is not an object of concern since it is the technical maneuver conversion that should always be used if there is a technical difficulty, anatomical doubt or surgical complications, since the surgeon returns to a more familiar and predictable environment. Yamazaki et al reported in a meta-analysis of 102 articles, conversion rates for multiportal operation of 7.2% and conversion to laparotomy in 1.69%^30^.

In the present study it becomes evident that the learning curve has a positive impact on the results, reducing to zero the conversion rate after the hundredth case. Even so, the safety of the procedure should always be the priority and the surgeon should not concern to converting to MPLS or laparotomy.

The mean surgical time observed was approximately 68 min and is within the mean found in the literature for MPLS and single port cholecystectomies, evidencing that the single port operation did not cause any impairment in relation to anesthetic time, increase of drugs used, longer time on surgery room and greater risk associated to the surgical time^1^ ^,^ ^3^ ^,^ ^4^ ^,^ ^11^ ^,^ ^13^ ^,^ ^16^ ^,^ ^18^ ^,^ ^21^ ^,^ ^26^ ^,^ ^27^.

In the present series it was observed that after the hundredth case there was a reduction of approximately 10 min in mean time, demonstrating that the learning curve represents an important variable in the search for better results^22^. Different devices of access to the cavity were used according to the technological evolution presented by the companies. During the study, almost all the approved devices in Brazil to single port were used, but the most frequent types were the SILS^®^ and Gelpoint^®^. Several reasons motivate the frequent use of these two brands like: ease to be use, greater triangulation of the laparoscopic instruments, possibility of introduction of laparoscopic instruments of various calibers, possibility of introduction of surgical gauze, help in extracting and protecting the skin^30^.

The use of conventional laparoscopic instruments was due to the availability, usuality and no additional cost, compared to the curved instruments. The study by Antoniou et al. with 1737 patients showed an increase in surgical time with the use of curved instruments in 32 min without showing any safety damage and corroborated our choice^2^.

In relation to surgical complications, pain in the incision pain and hematomas, seven cases were observed and all with very fast spontaneous resolution. The presence of granulomas were observed in five cases and motivated the exchange of polypropylene closure suture for polyglactin 910; granulomas stopped being a problem. In addition, it was found that they occurred in the majority of patients in leaner patients - mean BMI of 23.2 vs. without granulomas of 27.6 (p=0.041). This can be explained by the lower thickness of subcutaneous generating suture exteriorization. For cases that presented granulomas, they were removed using local anesthesia and small surgical exploration with resolution of 100% of the cases. These minor complications were found with values ​​very close to that found in the literature^11^ ^,^ ^16^ ^,^ ^29^ ^,^ ^30^.

The onset of herniations at the surgical site occurred in five cases (2.95%), a rate compatible with the literature and occurred in the population with a BMI >24 (p=0.004), with a mean BMI of 31.4 while the population had a mean BMI of 26.4. This fact is also found in the literature, which led to the preventive use of mesh in patients with a high risk of incisional herniation^18^ ^,^ ^20^ ^,^ ^27^ ^,^ ^29^ ^,^ ^30^.

This global herniation rate goes against the arguments that single-port access, by generating greater openness in the aponeurosis, could cause more hernias, which was not evidenced in this study and in another^24^ that found three times more chances of MPLS hernias in single-site operation. It is believed that this fact occurs because the surgeon ends up performing umbilical closure in the single port with direct vision due to having a larger incision size compared to MPLS^27^.

After identification of the hernias, they were electively corrected, with patient re-hospitalization and mesh use. No new recurrence was found.There was an isolated case of bile duct injury with partial section of the common bile duct probably due to local chronic inflammation; the conversion to MPLS did not add surgical advantage, which led to the conversion to laparotomy^1^ ^,^ ^13^ ^,^ ^26^.

## CONCLUSION

Single-port cholecystectomy can, after technical standardization and training of the surgical team, be a safe surgical procedure associated with the best aesthetic aspect of the operation.
